# *In vitro* modeling and rescue of ciliopathy associated with *IQCB1/NPHP5* mutations using patient-derived cells

**DOI:** 10.1016/j.stemcr.2022.08.006

**Published:** 2022-09-08

**Authors:** Kamil Kruczek, Zepeng Qu, Emily Welby, Hiroko Shimada, Suja Hiriyanna, Milton A. English, Wadih M. Zein, Brian P. Brooks, Anand Swaroop

**Affiliations:** 1 Neurobiology, Neurodegeneration & Repair Laboratory, National Eye Institute, National Institutes of Health, MSC0610, 6 Center Drive, Bethesda, MD 20892, USA; 2 Ocular Gene Therapy Core, National Eye Institute, National Institutes of Health, Bethesda, MD 20892, USA; 3 Ophthalmic Genetics and Visual Function Branch, National Eye Institute, National Institutes of Health, Bethesda, MD 20892, USA

**Keywords:** patient-derived iPSC, retinal organoids, photoreceptor, primary cilia, gene therapy, vision, Leber congenital amaurosis, Senior-Løken syndrome, stem cells, RPE

## Abstract

Mutations in the IQ calmodulin-binding motif containing B1 (*IQCB1*)*/NPHP5* gene encoding the ciliary protein nephrocystin 5 cause early-onset blinding disease Leber congenital amaurosis (LCA), together with kidney dysfunction in Senior-Løken syndrome. For *in vitro* disease modeling, we obtained dermal fibroblasts from patients with NPHP5-LCA that were reprogrammed into induced pluripotent stem cells (iPSCs) and differentiated into retinal pigment epithelium (RPE) and retinal organoids. Patient fibroblasts and RPE demonstrated aberrantly elongated ciliary axonemes. Organoids revealed impaired development of outer segment structures, which are modified primary cilia, and mislocalization of visual pigments to photoreceptor cell soma. All patient-derived cells showed reduced levels of CEP290 protein, a critical cilia transition zone component interacting with NPHP5, providing a plausible mechanism for aberrant ciliary gating and cargo transport. Disease phenotype in NPHP5-LCA retinal organoids could be rescued by adeno-associated virus (AAV)-mediated *IQCB1/NPHP5* gene augmentation therapy. Our studies thus establish a human disease model and a path for treatment of NPHP5-LCA.

## Highlights

• *NPHP5*-LCA patient-derived fibroblasts and RPE display abnormally elongated cilia
• Outer segment protein localization is impaired in patient-derived photoreceptors
• CEP290 protein reduction is observed across all *NPHP5*-LCA patient-derived cells
• *NPHP5* augmentation improves disease phenotypes in patient retinal organoids

## Graphical abstract

**Figure:**
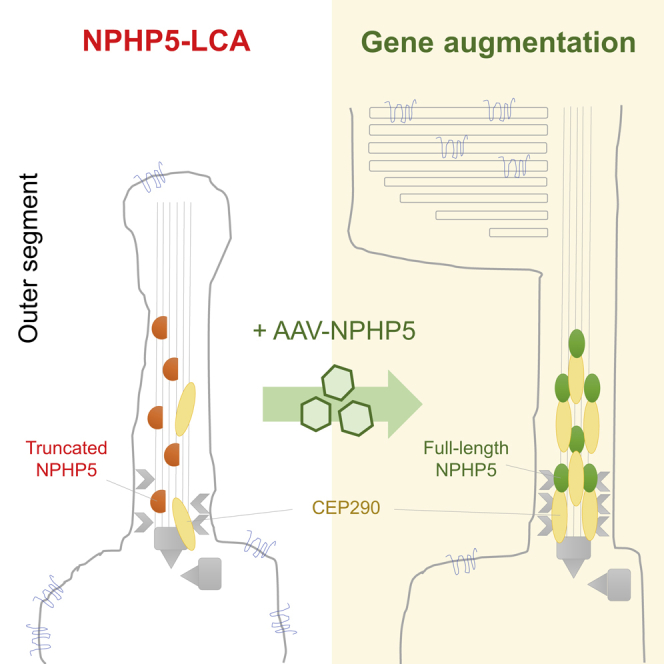

## Teaser

Swaroop and colleagues use *in vitro* disease modeling to characterize cilia abnormalities in human *NPHP5*-LCA patient-derived cells. Short photoreceptor outer segments with mislocalized opsin proteins and reduced CEP290 protein levels were observed in patient-derived retinal organoids. *NPHP5*-targeted gene therapy rescued the disease phenotypes in organoids, suggesting a key role of NPHP5 in stabilizing the CEP290-associated ciliary gate complex to ensure proper protein trafficking.

## Introduction

Once considered vestigial, the non-motile primary cilium has emerged as a key organelle in highly specialized sensory signal transduction in most eukaryotic cells (Breslow and Holland, 2019; Reiter and Leroux, 2017; Sang et al., 2011). Mutations in genes associated with cilia biogenesis and/or function lead to diseases termed ciliopathies, which encompass a plethora of varying phenotypes including developmental malformations, kidney cysts, intellectual disability, and sensory dysfunctions (Reiter and Leroux, 2017). In vertebrate photoreceptors, primary cilia acquire a unique architecture consisting of an axoneme surrounded by ciliary membrane with exquisitely organized stacks of discs that are critical for high-efficiency photon capture and signal transmission. Thus, cilia formation and functional maintenance are critical for vision, and photoreceptor defects and/or degeneration are commonly associated with ciliopathies (Chen et al., 2019).

Mutations in the gene encoding IQ calmodulin-binding motif containing B1 (*IQCB1*/*NPHP5*) are the most common cause of renal-retinal Senior-Løken syndrome (SLSN) (Otto et al., 2005) and are also detected in patients with non-syndromic Leber congenital amaurosis (LCA) (Estrada-Cuzcano et al., 2011; Stone et al., 2011). Both SLSN and LCA are genetically and clinically heterogeneous. Patients with LCA exhibit an early onset of retinal photoreceptor dysfunction that is accompanied by nystagmus, photophobia, and other clinical findings, whereas SLSN additionally includes nephronophthisis (NPHP), a kidney cystic disease leading to chronic renal failure (Hildebrandt et al., 2009). Notably, patients with SLSN exhibit variable onset of kidney dysfunction, whereas visual function defects are observed in early childhood and have a significant impact on patients’ quality of life, making photoreceptors an important target for therapy development.

NPHP5 protein of 598 amino acids is required for cilia biogenesis and interacts with multiple cilia-associated proteins including RPGR (Otto et al., 2005) and CEP290 (NPHP6) (Barbelanne et al., 2013; Schafer et al., 2008). NPHP5 is localized in the ciliary transition zone with CEP290, where it modulates the integrity of the BBSome protein complex involved in ciliary transport (Barbelanne et al., 2015), and the assembly of basal feet during cilia formation (Hossain et al., 2020). Of particular interest is CEP290, since patients with LCA carrying *NPHP5* or *CEP290* mutations exhibit overlapping clinical phenotypes (Cideciyan et al., 2011; Otto et al., 2005), suggesting involvement in similar functions within cilia. CEP290, however, interacts with many proteins, exhibits domain-specific functions, and is associated with a broader range of ciliopathies (Coppieters et al., 2010; Drivas and Bennett, 2014; Rachel et al., 2012, 2015). Patient mutations in *IQCB1* cluster around the coiled-coil domains in the C-terminal region, with the SLSN-associated mutations leading to an earlier truncation of the protein than those responsible for LCA (Estrada-Cuzcano et al., 2011). Retinal imaging in patients with NPHP5-LCA reveals rapid and widespread rod photoreceptor degeneration but relatively preserved central region of non-functional cone photoreceptors likely lacking outer segments (Cideciyan et al., 2011; Downs et al., 2016). These observations suggest that the remaining cones could be a viable target for gene replacement therapy (Cideciyan et al., 2011).

Loss of function of *Nphp5* in mouse and dog models results in impaired outer segment formation, absence of rod or cone responses on electroretinogram, and early-onset rod photoreceptor loss (Downs et al., 2016; Ronquillo et al., 2016). These models have been used for *in vivo* assessment of prospective therapies (Aguirre et al., 2021; Hanke-Gogokhia et al., 2018). However, animal models do not completely recapitulate human genetic diversity and features of retinal development (Hoshino et al., 2017; Yan et al., 2020). Furthermore, efficacy of transduction with gene therapy vectors differs between model organisms and human retina (Gonzalez-Cordero et al., 2018; Wiley et al., 2018). Retinal organoids derived from patient induced pluripotent stem cells (iPSCs) complement the *in vivo* studies using animal models by providing a human genetic context (Kruczek and Swaroop, 2020).

Retinal organoids are *in vitro* self-formed aggregates from PSCs displaying many key characteristics of the native tissue (Nakano et al., 2012; Zhong et al., 2014). To model genetic heterogeneity of LCA, elucidate disease mechanisms, and evaluate possible treatment paradigms, we recently established retinal organoid culture systems from iPSCs of patients with LCA with mutations in *CEP290* and *CRX* (Kruczek et al., 2021; Shimada et al., 2017). Here, we report the phenotypic modeling of NPHP5-LCA using patient dermal fibroblasts, iPSC-derived retinal pigment epithelium (RPE) cells, and retinal organoids, which exhibit abnormal cilia morphology and reduced levels of CEP290. The photoreceptors in patient organoids demonstrate impaired protein localization and aberrant extension of outer segments. We also show that this phenotype can be rescued by adeno-associated virus (AAV)-mediated delivery of the correct *IQCB1/NPHP5* sequence. Together with the animal models (Aguirre et al., 2021; Hanke-Gogokhia et al., 2018), our studies validate gene augmentation as a prospective treatment approach for NPHP5-LCA.

## Results

### Patients and experimental design

**Figure 1:**
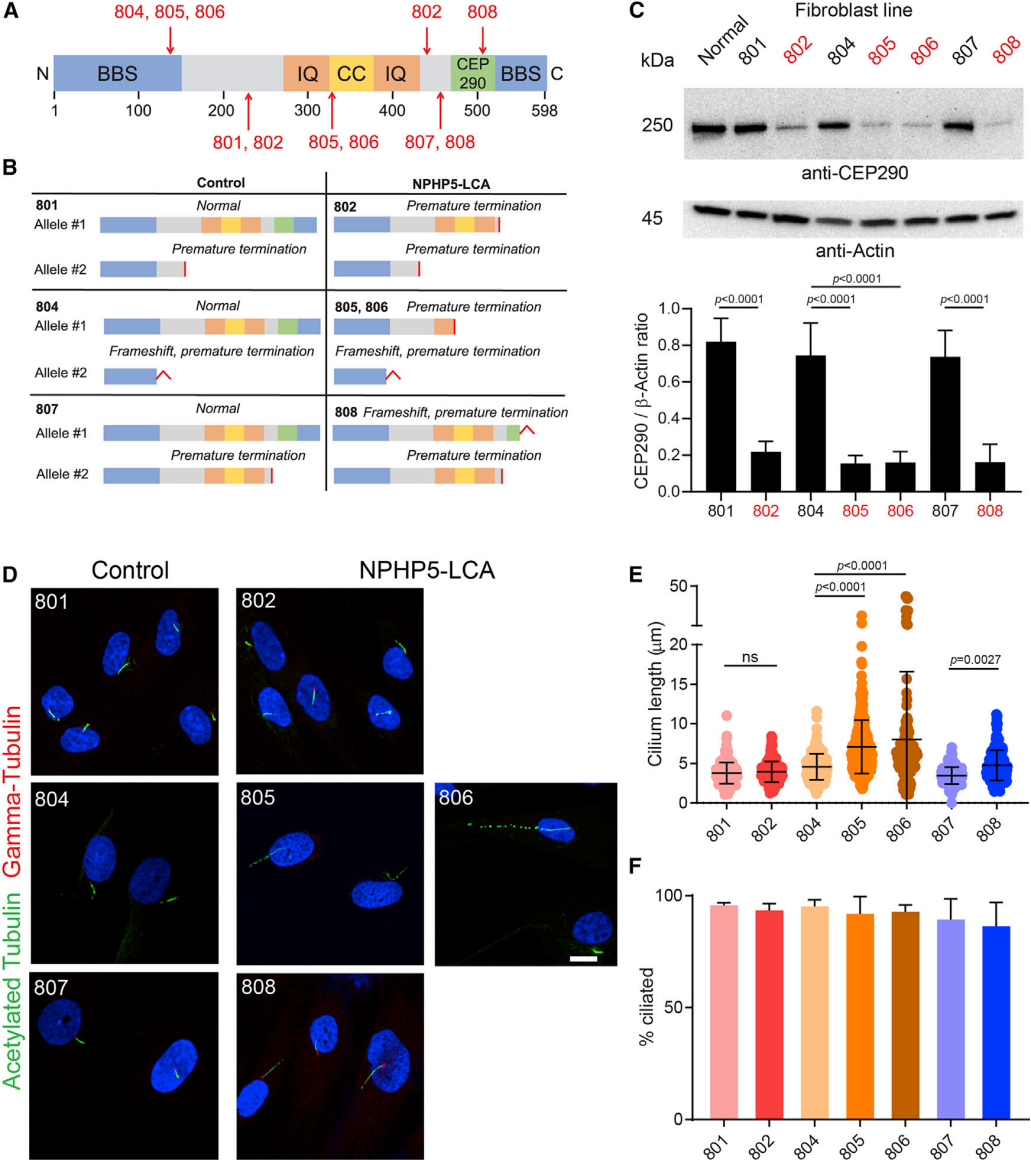
NPHP5-LCA mutations affect cilia length in patient fibroblasts (A) A schematic of the NPHP5 protein (598 aa), showing functional domains and location of mutations (numbers in red). BBS, BBSome binding region; IQ, IQ-calmodulin binding motif; CC, coiled-coil domain; CEP290, CEP290 binding region. (B) A table representing combinations of predicted mutant NPHP5 protein products in patient and familial control subjects. (C) Immunoblot analysis of protein extracts from fibroblast lines using CEP290 antibody. Note substantial reduction of CEP290 protein in patient lines (indicated in red). Actin was used as a loading control. A representative immunoblot of 3 independent replications is shown here. Densitometry quantification of all experiments is presented in the bottom panel, and p values from one-way ANOVA are indicated. (D) Immunocytochemistry of control and patient fibroblasts stained with γ-tubulin (basal body marker) and acetylated tubulin (axoneme marker) antibodies. Scale bar, 5 μm. (E) Cilia length determined by measuring acetylated tubulin staining. Cilia in 805 and 806 are significantly longer compared with all familial controls (801, 804, and 807). Cilia in 808 show a significant difference compared with the familial control (807) but not the unrelated controls 801 and 804. Median ± SD; number of cilia counted for 801 n = 233, 802 n = 221, 804 n = 227, 805 n = 268, 806 n = 155, 807 n = 190, and 808 n = 195; data are from 2 (lines 801–806) or 3 (lines 807 and 808) independent replication experiments; and p values from one-way ANOVA test are indicated. (F) The number of cilia, determined through quantifying γ-tubulin and acetylated tubulin staining. Mean ± SD are plotted. No significant changes found across lines.

To examine ciliopathy phenotypes associated with mutations in the *IQCB1* gene causing NPHP5-LCA, we recruited 4 affected patients as well as 3 healthy familial controls from 3 families (Tables S1 and S2; Figure S1). These individuals carried 6 different mutant alleles across the *IQCB1/NPHP5* coding sequence (Figures 1A and 1B; Table S1). Unaffected carriers (controls) carry a single mutant allele in a heterozygous state, whereas patients are compound heterozygotes for two mutant alleles (Figure 1B; Table S1). Three of the identified mutations are present in known protein domains: (1) c.421_422delTT (p.F141fsX6) localizes to the N-terminal region containing the BBS interaction site (1–157 amino acids), (2) c.1036G>T (p.E346X) occurs within a coiled-coil domain (340–373 amino acids), and (3) c.1516_1517delCA (p.H506fsX13) is present just before the CEP290 binding region (509–529 amino acids) (Figure 1A). We obtained skin biopsies from these patients to model disease pathology and evaluate treatment strategy.

### Elongated cilia morphology in dermal fibroblasts from patients with NPHP5-LCA

Given the relationship with NPHP5, we first examined CEP290 protein in fibroblasts by immunoblotting. Significantly reduced amounts of CEP290 were evident in all patient samples (Figure 1C; p < 0.0001). To assess how the mutations impact ciliogenesis, we subjected patient and familial control fibroblasts to serum starvation for 72 h and quantified cilia length and incidence by immunohistochemistry using anti-acetylated tubulin (axoneme) and anti-γ-tubulin (basal body) antibodies (Figure 1D). We observed strikingly elongated cilia in two sibling patients of the same genotype (805 and 806; Figure 1E; p < 0.0001). Cilia in fibroblasts from another patient (808; Figure 1E) showed increased length relative to familial control (807; p = 0.0027) but not compared with the other two unrelated controls (801 and 804). Notably, these 3 patients carry mutations that localize to known functional domains of NPHP5 (see Figure 1A). The number of basal bodies per cell across all samples showed no significant difference (Figure 1F), suggesting that initial steps in cilia formation were not affected. In conclusion, NPHP5-LCA mutations in known functional protein domains influenced cilia length in patient dermal fibroblasts.

### Cilia defects in RPE cells derived from iPSCs of patients with NPHP5-LCA

**Figure 2:**
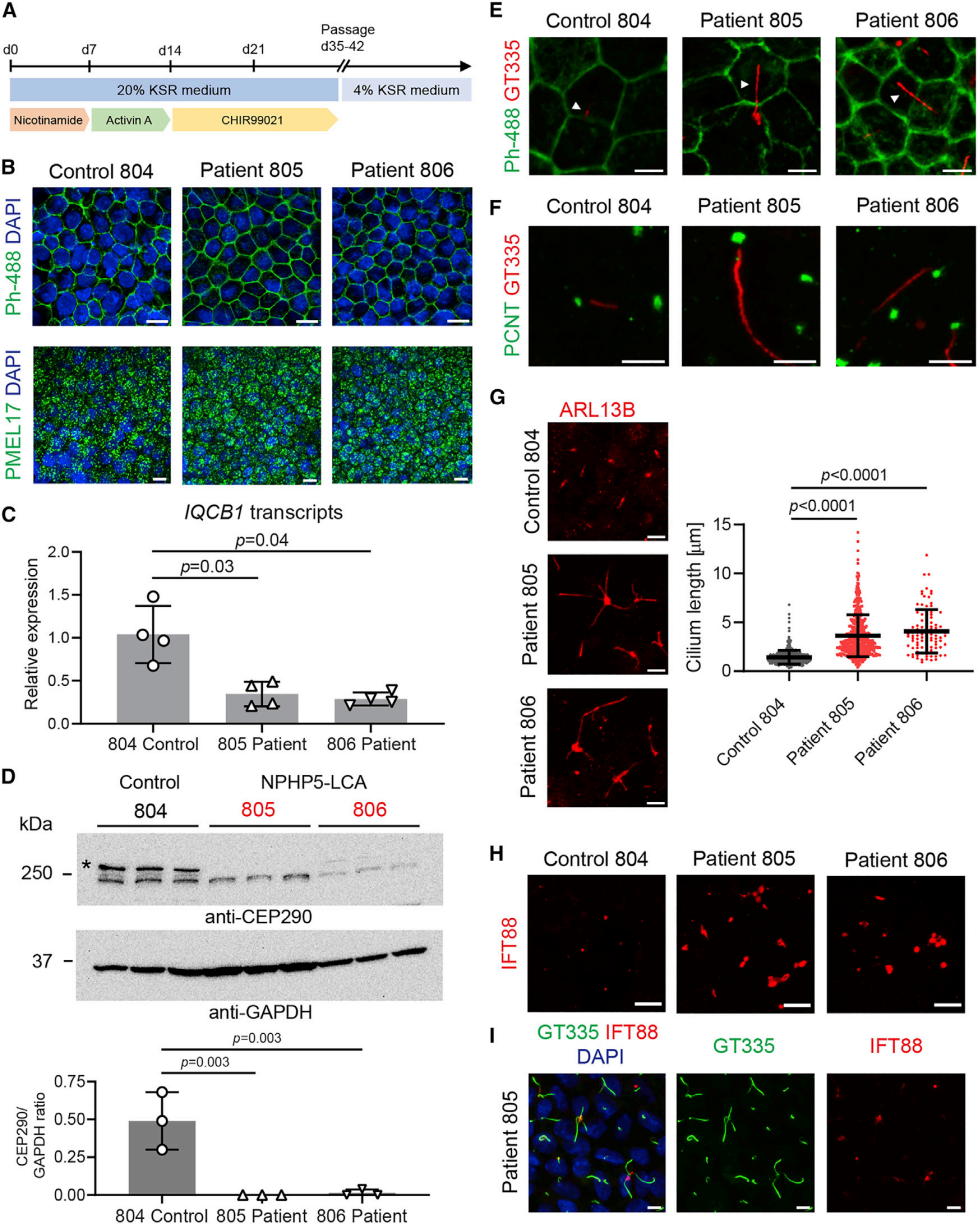
Abnormal cilia morphology in RPE cells from NPHP5-LCA patient (A) A schematic representation of the RPE differentiation protocol used in the study. (B) Immunostaining of control and patient RPE cells at week 10 of differentiation using phalloidin conjugated to Alexa 488 (top panels, Ph-488) and PMEL17 antibody (bottom panels). Nuclei are visualized with DAPI. Scale bars, 10 μm. (C) Quantitative PCR detection of *IQCB1* transcripts in RNA samples from control and patient iPSC-derived RPE cells. Two replicate samples from 2 independent differentiation cultures were analyzed in triplicates, and p values are from one-way ANOVA. (D) Immunoblot analysis using the CEP290 antibody. GAPDH served as loading control. Note clearly lower levels of CEP290 in patient samples (red labels). A single immunoblot is shown using protein extracts from 3 independent batches of RPE differentiation. Densitometry quantification of the upper band of correct size labeled with asterisk is presented in lower panel, and statistical analysis was performed using ANOVA. (E) Actin-rich cell junctions labeling using Alexa 488-conjugated phalloidin combined with GT335 immunostaining to visualize elongated cilia in patient samples in the context of cell morphology. Scale bars, 5 μm. (F) Control and patient-derived iPSC-RPE co-immunostained for basal body marker pericentrin (PCNT) and polyglutamylated tubulin within ciliary axonemes (GT335). Scale bars, 5 μm. (G) Immunostaining for ARL13B demonstrates altered axonemal morphologies. Scale bars, 5 μm. Quantification of cilia length; median ±SD; N = 3 experimental replications using independent differentiation batches. n = 497 individual cilia measured in control, n = 529 cilia in patient 805, and n = 97 in patient 806 samples; p values are from one-way ANOVA. (H) Immunostaining for IFT88. Accumulations of IFT88 are detected in patient RPE. Representative images are shown from 3 experimental replications using independent batches of cells. Scale bar, 10 μm. (I) IFT88 immunostaining in patient 805 iPSC-RPE combined with GT335 and DAPI. Scale bars, 5 μm.

We selected the two patients with the strongest cilia phenotype in fibroblasts (805 and 806) as well as the corresponding familial control (804) for reprogramming into iPSCs. The resulting lines had the colony morphology typical of stem cells (Figure S2A), harbored original patient mutations (Figure S2B), were of normal karyotypes (Figure S2C), and expressed pluripotency markers (Figures S2D and S2E). A report of RPE defects preceding photoreceptor degeneration in a mouse model of BBSome-associated ciliopathy (May-Simera et al., 2018) prompted us to differentiate iPSCs into RPE using a recent protocol (Regent et al., 2020), which is schematically depicted in Figure 2A. RPE cells from control and patient lines were morphologically similar with a characteristic cobblestone appearance (Figure 2B) and expressed typical RPE markers such as PMEL17 (Figure 2B). Levels of *NPHP5* mRNA were significantly reduced in 805 and 806 patient iPSC-derived RPE (Figure 2C; p = 0.03 and 0.04), suggesting potential degradation of mutant transcripts. As in the case of dermal fibroblasts, CEP290 protein was clearly reduced in RPE cells differentiated from patient iPSCs (Figure 2D, asterisk denotes the correct protein band). Immunostaining of cilia basal body marker, pericentrin (PCNT), polyglutamylated tubulin present in ciliary axoneme (with GT335 antibody), and cilia membranes with ARL13B antibody revealed abnormal and strikingly elongated cilia morphology in RPE derived from the two patients with NPHP5-LCA (Figures 2E–2G). While average cilium length in control RPE cells was 1.4 μm (n = 497 individual cilia, N = 3 experiments using independent differentiation batches), patient cilia were significantly longer with a mean of 3.6 μm for patient 805 (n = 529, N = 3) and 4.1 μm for patient 806 samples (n = 97, N = 3, Figure 2G; p < 0.0001 in both comparisons with control, one-way ANOVA). NPHP5 directly interacts with BBSome, which regulates ciliary traffic (Barbelanne et al., 2015). Consistent with the loss of one of the BBSome interaction sites in the predicted mutant NPHP5 protein in patients 805 and 806, we identified enhanced accumulation of intraflagellar transport protein, IFT88, in patient cilia compared with the familial control cells (Figures 2H and 2I). Thus, patient-derived RPE cells show morphological abnormalities of primary cilia.

### Serum-free conditions for studying cilia and photoreceptor segments in retinal organoids

**Figure 3:**
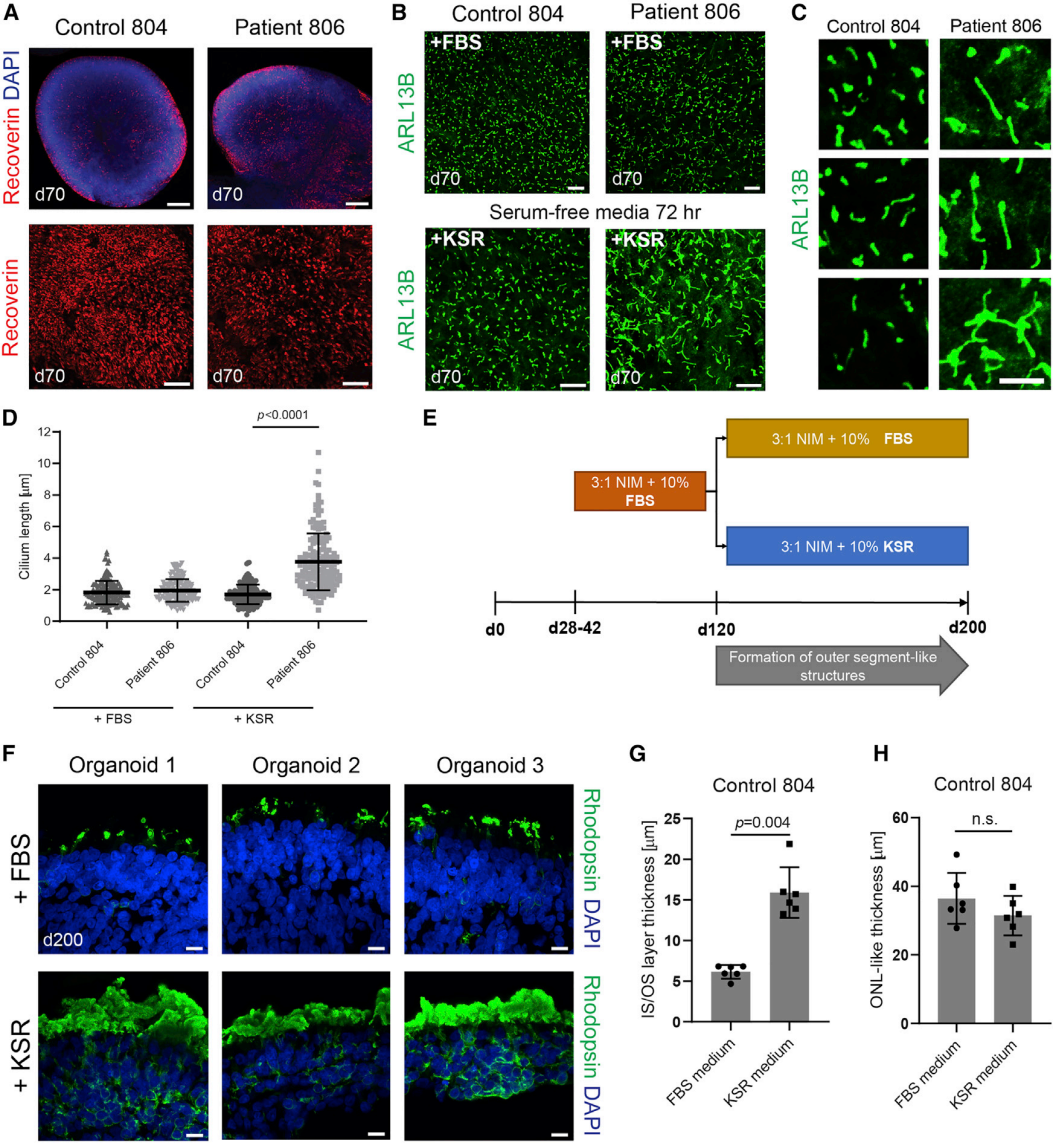
Effect of serum-free culture conditions on cilia formation and differentiation in retinal organoids (A) Whole-mount immunostaining for the photoreceptor marker recoverin at day 70, showing developing photoreceptor cells on the external apical surface of organoids. Scale bars for top panels, 200 μm, and for bottom panels, 50 μm. (B) Whole-mount immunostaining for the ciliary membrane marker ARL13B in organoids, cultured in regular FBS-containing media or media supplemented with KSR for 72 h. Note extended cilia in patient organoid in serum-free condition. Scale bars, 10 μm. (C) Enlarged areas showing cilia morphology in serum-free condition. Scale bar, 5 μm. (D) Quantification of cilia length the two media conditions; median ± SD; N = 3 organoids; n = 150 total cilia in each group; p < 0.0001. Welch’s t test was used to compare control and patient samples. (E) Schematic of serum-free media testing experiment. FBS was replaced with KSR, which is a defined mix without mitogens. (F) Immunostaining of rhodopsin at day 200 of differentiation in control (804) organoids cultured in FBS- or KSR-supplemented media. Scale bar, 10 μm. (G and H) Quantification of outer nuclear layer-like thickness and inner/outer segment layer thickness. n = 6 organoids in each group; mean ± SD is plotted; p value is from Student’s t test. Also see Figure S3.

Following our previously published protocols (Kaya et al., 2019; Kelley et al., 2020), we generated retinal organoids from both control and patient iPSC lines (Figure 3A). To promote ciliogenesis in these retinal organoid cultures, we substituted serum (FBS) in our original protocol with serum replacement supplement (KSR), which does not contain lysophosphatidic acid, a phospholipid that has been reported to block ciliogenesis via the Akt signaling pathway (Walia et al., 2019). Initial experiments on early-stage retinal organoids (day 70) cultured with KSR-supplemented media for 72 h demonstrated elongated cilia compared with serum conditions (Figure 3B). Notably, cilia in serum-free-cultured patient-derived organoids at this stage were significantly longer when compared with equivalent control organoids (Figures 3C–3D; p < 0.0001). To better target photoreceptor cilia in subsequent experiments, KSR was substituted from day 120 onwards, when photoreceptor outer segments begin to form in organoids (Capowski et al., 2019; Kaya et al., 2019) (Figure 3E). As a proof of concept, we demonstrate that late stage (day 200) control organoids cultured with KSR serum-free conditions have significantly increased inner segment/outer segment (IS/OS) thickness, indicating longer photoreceptor cilia, compared wiht organoids cultured with FBS (Figures 3F and 3G; p = 0.004). Importantly, photoreceptor differentiation in control organoids was comparable with this modification without affecting ONL thickness (Figure 3H, p = not significant [n.s.]), and serum-free medium supported long-term maintenance of organoids up to 1 year in culture (Figure S3).

### Photoreceptor disease phenotype in NPHP5-LCA retinal organoids

**Figure 4:**
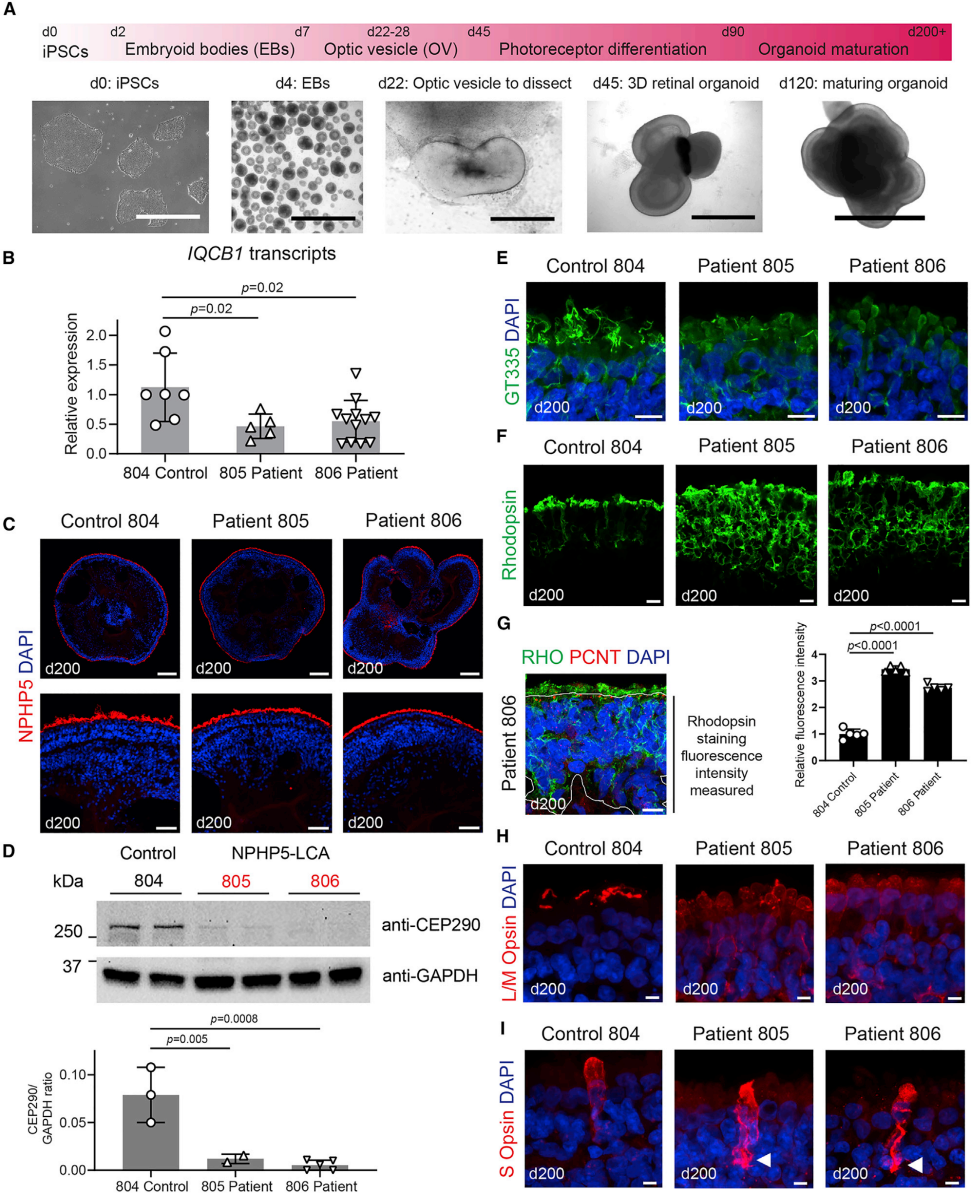
Mutations in NPHP5 lead to diminished CEP290 protein levels and rhodopsin mislocalization in retinal organoids (A) An overview of retinal organoid differentiation protocol. Scale bars, 1,000 μm at days 0, 4, and 45 and 400 μm at days 22 and 120. (B) Quantitative PCR detection of *IQCB1* transcripts in RNA samples from control and patient retinal organoids. Five to twelve RNA samples of pooled 8–12 individual organoids from 2–5 independent differentiation cultures were analyzed in triplicates. p values are from one-way ANOVA. Mean ± SD are plotted. (C) NPHP5 immunostaining in control and NPHP5-LCA patient-derived retina organoids at day 200. Scale bars for the top panels, 200 μm, and for the bottom panels, 50 μm. (D) Representative immunoblot with CEP290 antibody using protein extracts from control and patient organoids collected at day 200. GAPDH used as loading control. Note clear reduction in CEP290 protein in patient samples (labeled in red). Immunoblotting was performed twice using samples from 2–5 independent differentiation batches (10–12 organoids per sample). Densitometry quantification results are presented in the bottom panel, and statistical analysis was performed using ANOVA. Mean ± SD are plotted. (E) Staining of polyglutamylated tubulin, a marker of connecting cilium microtubules. Note diminished staining in patient samples. Scale bar, 10 μm. (F) Immunostaining of rhodopsin in control and patient organoids at day 200. Scale bar, 10 μm. Rhodopsin is visibly mislocalized to rod cell somas in patient samples. (G) Quantification of rhodopsin staining in cell somas. Image on the left shows an example of the area selected for measurements. Fluorescence measurements normalized to control organoids were obtained from data of 5 individually cultured organoids per group, and 3 sections averaged each, differentiated at the same time in a single use. p value from one-way ANOVA analysis. Mean ± SD are plotted. (H and I) Cone opsin staining in control and NPHP5-LCA patient organoids at day 200. L/M-opsin shows a diffuse pattern in cell soma in patient samples compared with distinct localization to outer segments in control (H). S cones display similar morphology, but S-opsin tends to be mislocalized to axons and synaptic pedicles in NPHP5-LCA patient organoids (I). Scale bar, 5 μm. Also see Figures S4 and S5.

Both control and patient iPSCs were able to generate retinal organoids (Figure 4A) with typical VSX2+ neuroepithelia at early stages of differentiation (Figure S4A) and neuroepithelial lamination at later stages of cultures, with CRX+ (Figure S4A) and recoverin+ (Figures S4A–S4C; Figure 3A) photoreceptor cells aligning at the apical edge of the epithelium and BRN3A+ retinal ganglion cells present at the basal side (Figure S4A). *IQCB1/NPHP5* transcripts showed reduced levels in mature patient organoids at day 200 (Figure 4B). The NPHP5 protein localized to the apical edge, where cilia/OSs are present in organoids derived from control and patient iPSCs (Figure 4C). Similar to dermal fibroblasts and RPE, CEP290 levels were significantly reduced in protein extracts from NPHP5-LCA patient organoids compared with the familial control (Figure 4D; p ≤ 0.005). Glutamylated tubulin (GT335) showed diminished and diffuse patterns of staining in patient organoids compared with control samples (Figure 4E), suggesting that the connecting cilia (and consequently OSs) formation was compromised. Notably, we detected mislocalization of rhodopsin to the cell soma instead of the rod OSs in patient-derived organoids (Figure 4F). Quantification of rhodopsin showed almost 3× higher levels of fluorescence intensity in the outer nuclear layer of organoid photoreceptors from the two patients compared with the familial control (Figure 4G; n = 5 organoids per group from 2 independent batches, 3 sections examined from each organoid, p *<* 0.0001, one-way ANOVA). Overall, levels of rhodopsin protein as detected by immunoblotting showed a trend for increased amount in patient organoids (Figure S5A), consistent with the pronounced mislocalization to rod cell bodies observed with immunostaining. While total L/M-opsin protein levels showed higher levels of variability in control and patient-derived organoids (Figure S5B), L/M-opsin distribution showed diffuse labeling in the cell soma of patient organoids rather than a distinct localization to the OSs as observed in the control (Figure 4H). S-opsin-expressing cones exhibited similar morphologies; however, we frequently observed S-opsin mislocalization to axons and synaptic pedicles in patient organoids (Figure 4I, arrowheads; Figure S5C). Thus, retinal organoids from NPHP5-LCA iPSC lines demonstrated deficiencies in the development of photoreceptors consistent with the phenotype in animal models and clinical findings (Cideciyan et al., 2011; Downs et al., 2016; Ronquillo et al., 2016).

**Figure 5:**
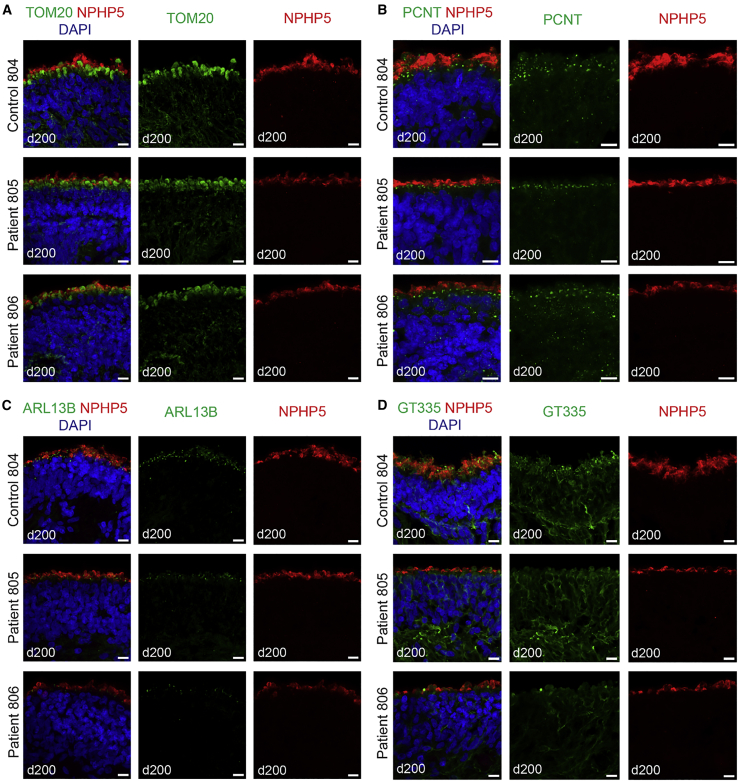
Localization of NPHP5 protein in control and patient retinal organoids Immunostaining of cryosections of control or NPHP5-LCA patient retinal organoids collected at day 200 of differentiation using NPHP5 antibody and a set of antibodies specific to inner segment and cilia proteins. (A–D) TOM20, a mitochondrial protein enriched in inner segments of photoreceptors (A), PCNT, a protein associated with cilia basal bodies (B), ARL13B, a marker of ciliary membrane (C), and polyglutamylated tubulin (GT335), which marks stable microtubules present in the ciliary axoneme (D). NPHP5 localizes primarily distal to inner segments marked with TOM20 and basal bodies labeled with PCNT, but it partially overlaps with ARL13B staining of ciliary membrane and polyglutamylated microtubules beyond the tips of photoreceptor inner segments. Scale bars, 10 μm.

We also noted that NPHP5 was detected predominantly distal to photoreceptor ISs (TOM20; Figure 5A) and in cilia basal bodies (PCNT; Figure 5B) but partially overlapped with the cilia membrane (ARL13B; Figure 5C) and connecting cilia (GT335; Figure 5D). Immunostaining for all of these proteins was reduced in patient organoids, with a more dramatic decrease in patient 806 organoids. These results indicate that *IQCB1/NPHP5* mutation(s) impact proper localization of cilia proteins, resulting in defective photoreceptor OS morphogenesis.

### Rescue of photoreceptor defects with AAV-mediated *NPHP5* gene replacement

**Figure 6:**
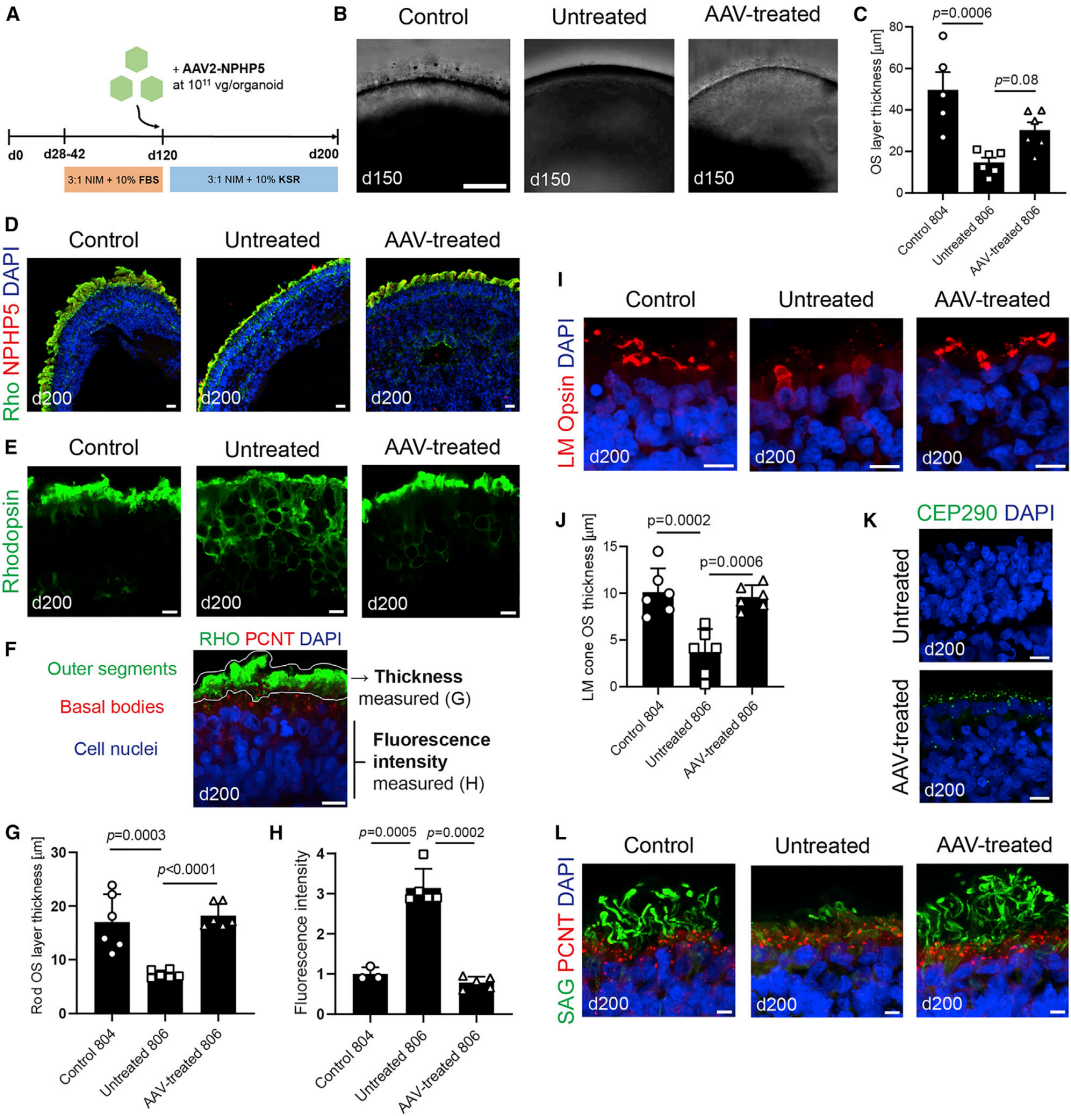
AAV-mediated *NPHP5* gene replacement rescues photoreceptor outer segment defects in retinal organoids (A) Schematic of the timeline of the gene therapy experiment. (B) Bright-field images of organoids at day 150. Scale bar, 100 μm. (C) Quantification of OS brush-like protrusion thickness, and p values are from one-way ANOVA. Mean ± SD are plotted. (D and E) Immunostaining for rhodopsin in control, untreated (806 line), and AAV-treated organoids. Scale bars, 10 μm. (F) A representative image indicating areas used for measurements and corresponding cellular structures. (G) Quantification of rod inner/outer segment thickness; mean ± SD; N = 2 independent experiments, 6 organoids each group (3 per experiment). p values are from one-way ANOVA. (H) Quantification of rhodopsin mislocalization to cell soma; N = 2 independent experiments, 3–5 organoids per group. p values are from one-way ANOVA. Mean ± SD are plotted. (I) Immunostaining for L/M cone opsins. Scale bar, 20 μm. (J) Quantification of L/M cone outer segment layer thickness; mean ± SD; N = 2 independent experiments, 6 organoids each group (3 per experiment). p values are from one-way ANOVA. (K) Immunostaining of CEP290 in untreated and AAV-treated 806 patient retinal organoids. Scale bars, 10 μm. (L) Immunostaining for visual arrestin (SAG) and PCNT further demonstrating restoration of outer segment formation following AAV treatment. Scale bar, 5 μm. Also see Figure S6.

We then made an AAV vector containing the correct copy of the human *IQCB1/NPHP5* coding sequence under the control of a cytomegalovirus (CMV) promoter. The vector was packaged into an AAV2 serotype capsid. *NPHP5* transgene was efficiently expressed from this vector when added to patient organoids at d120 (Figure 6A; Figures S6A and S6B). Vector transduction partially restored the formation of light-reflective “brush,” corresponding to developing photoreceptor OSs, on the apical surface of organoids at day 150 (Figures 6B and 6C). In day 200 organoids, rod OS structures significantly increased in length following AAV-NPHP5 treatment (Figures 6D–6G; p *<* 0.0001), and the abnormal accumulation of rhodopsin in the somas of patient rods was abrogated following gene augmentation (Figures 6E–6H; p = 0.0002). L/M-opsin cone OSs also significantly recovered after AAV-NPHP5 treatment (Figures 6I and 6J; p = 0.0006), and CEP290 protein became detectable in the AAV-treated organoids (Figure 6K). The localization of other OS proteins, including visual arrestin (SAG; Figure 6L), peripherin2 (PRPH2; Figure S6C), phosphodiesterase 6B (PDE6B; Figure S6D), and rod α-transducin (GNAT1; Figure S6E), was restored to varying degrees after AAV transduction. We did not detect significant changes in OS gene expression across control, patient, and AAV-NPHP5-treated organoids (Figure S6F), suggesting that augmented expression of NPHP5 in patient-derived photoreceptors specifically improves OS protein localization. While S-opsin-expressing cones showed similar morphologies, we detected a reduced level of S-opsin mislocalized to axons and synaptic pedicles following the AAV treatment (Figure S6G). Together, these data suggest that OS biogenesis was at least partially rescued in NPHP5-LCA patient organoids following AAV-mediated gene augmentation.

## Discussion

Defects in assembly, trafficking, and/or signaling of primary cilium can lead to compromised function and, consequently, a wide range of clinical presentations, with photoreceptor degeneration being a frequently observed phenotype. Mutations in a number of cilia genes can lead to phenotypically heterogeneous retinal diseases, such as LCA and Bardet-Biedl syndrome (Chandra et al., 2021; Chen et al., 2021), with syndromic or non-syndromic manifestations. *IQCB1/NPHP5* gene mutations cause LCA and/or associated NPHP (Otto et al., 2005). Studies in cultured cells and animal models have provided insights into NPHP5 function during cilia formation and suggested avenues for treatment of NPHP5-LCA (Aguirre et al., 2021; Barbelanne et al., 2015; Hanke-Gogokhia et al., 2018; Hossain et al., 2020; Ronquillo et al., 2016). In this article, we demonstrate, using NPHP5-LCA patient-derived cells, the role of NPHP5 in cilia morphogenesis as well as validation of a potential gene therapy approach for correcting photoreceptor cilia defects in LCA.

NPHP5 is part of a larger protein interaction network, forming cilia gating complexes and integrating diverse signaling pathways (Rachel et al., 2012; Sang et al., 2011; Takao et al., 2017). All pathogenic truncating mutations in NPHP5 disrupt its binding with CEP290, thereby suggesting an important functional relevance of this interaction (Barbelanne et al., 2013, 2015; Sang et al., 2011; Schafer et al., 2008). Overlap of clinical findings further supports the significance of NPHP5-CEP290 interaction (Cideciyan et al., 2011). In patients with CEP290-LCA, the presence of residual CEP290 protein is proposed to be associated with a relatively milder phenotype, limited to vision loss and anosmia, in contrast to more severe pleiotropic phenotypes observed with other CEP290 mutations (den Hollander et al., 2006; Drivas et al., 2015; McEwen et al., 2007; Sayer et al., 2006). To our knowledge, this is the first report showing reduced levels of CEP290 protein in NPHP5-LCA patient-derived fibroblasts as well as in RPE and retinal organoids derived from patient iPSCs. We can therefore hypothesize that LCA caused by *IQCB1/NPHP5* mutations is the result of reduced CEP290 protein in sensory tissues and that NPHP5 is needed to stabilize the ciliary gate complex.

Knockdown of NPHP5 or CEP290 in cultured cells disrupts cilia formation by impacting the docking of basal bodies to the cell cortex (Barbelanne et al., 2013). In the *Nphp5*^*−/−*^ mouse retina, basal bodies are detected, but the distal transition zone is not fully developed in photoreceptors (Ronquillo et al., 2016). Notably, basal bodies are formed in both *Cep290*^*rd16/rd16*^ (a model of CEP290-LCA) and *Cep290*^*−/−*^ (a model of more severe Joubert syndrome) mouse models, with rudimentary cilia observed only in the milder *Cep290*^*rd16/rd16*^ retina (Rachel et al., 2015). Retinal organoids derived from iPSCs of patients with CEP290-LCA also exhibit photoreceptor cilia defects (Shimada et al., 2017). We predict that all of our NPHP5-LCA patient-derived cells express only the truncated NPHP5 protein, which lacks one BBSome interaction domain as well as the CEP290 binding domain. Clinical examination suggested a potentially more severe disease phenotype based on fundus autofluorescence imaging associated with mutations leading to shorter forms of NPHP5 (Figure S1; earlier truncations in 805/806 versus longer predicted fragments in 808; Figure 1B). In accordance, we detected more pronounced morphological changes in cilia of fibroblasts derived from these patients (Figures 1D and 1E). Our studies demonstrate that cilia are formed in these patient-derived cells, yet the ciliary axonemes are abnormal and the proteins that are targeted to the photoreceptor OS mislocalized. We can explain these observations by proposing aberrant ciliary gating and altered cargo transport due to reduced CEP290 levels as well as compromised control of BBSome-mediated ciliary traffic. The patient-specific retinal organoids, established in this study, should help in further dissection of NPHP5 functions in photoreceptor cilia.

Concordant with our hypothesis, a recent report shows that Rpgrip1l, another CEP290-interacting cilia protein, controls the amount of Cep290 at the transition zone and that loss of Rpgrip1l results in elongated cilia accompanied by diminished levels of Cep290 (Wiegering et al., 2021). Together with these observations, our study raises an interesting possibility that differential transport of cargo proteins at the ciliary gate is regulated by differing levels of CEP290 protein and associated complexes, which can be correlated with disease severity not only for CEP290 mutations (Parfitt et al., 2016; Shimada et al., 2017) but also for proteins in its network (such as NPHP5 or RPGRIP1L) (Wiegering et al., 2021; this work).

Integrity of the primary cilium is reported to be essential for RPE maturation, which in turn is necessary for photoreceptor function (May-Simera et al., 2018). Dysfunction of the RPE can lead to photoreceptor degeneration in retinopathies. *IQCB1/NPHP5* mutations are believed to primarily affect photoreceptor cells; nonetheless, we also detected cilia defects in patient iPSC-derived RPE, raising the possibility that incomplete maturation of RPE may also contribute to disease etiology.

Gene therapy using AAV vectors has emerged as an effective treatment approach for many inherited retinopathies (Trapani and Auricchio, 2018). Previous studies have demonstrated improved visual function in *Nphp5*^*−/−*^ mice and a spontaneous dog model carrying *NPHP5* mutation by using an AAV vector to deliver the complete *IQCB1/NPHP5* coding region (Aguirre et al., 2021; Hanke-Gogokhia et al., 2018). In this study, we were able to partially rescue OS development in NPHP5-LCA retinal organoids with improvements in both L/M cones as well as rod cells by AAV-mediated *IQCB1/NPHP5* gene augmentation. Treatment effect in L/M cones is particularly important given that useful high-acuity color vision in humans relies on cones and that cone cell bodies are present in the fovea of NPHP5-LCA patients at all ages studied (Cideciyan et al., 2011). The animal models provided essential *in vivo* proof of concept, whereas our work complements these studies with a human-specific system and evaluated the rescue of photoreceptor phenotype in patient-derived tissue *in vitro*.

To conclude, we used cells derived from patients with NPHP5-LCA to develop *in vitro* models to examine ciliopathy phenotypes. Reduced CEP290 protein as well as abnormal cilia morphology provided useful mechanistic insights into NPHP5-LCA pathogenesis. We could also rescue photoreceptor OS defects in retinal organoids by AAV-mediated gene therapy, highlighting the utility of this model system in developing future treatments for ciliopathies.

## Experimental procedures

### Culture of patient-derived cells

Skin biopsies were obtained after informed consent from 4 patients carrying *NPHP5* mutations and 3 familial healthy control subjects (Table S1). Dermal fibroblasts from subjects 804, 805, and 806 were reprogrammed into iPSCs using Sendai virus. Resulting iPSC lines were of normal karyotype, free of mycoplasma contamination, and maintained and expanded on Matrigel-coated dishes using E8 medium. ROCK inhibitor Y-27632 was used for splitting at 10 μM. Differentiation to RPE cells was performed as previously described (Regent et al., 2020). Retinal organoids were derived as described (Kaya et al., 2019), with the modification of replacing fetal bovine serum with KSR (10%) from day 120 onwards.

### Immunostaining

The method has been described previously (Shimada et al., 2017). A full list of antibodies is provided in Table S3. Imaging was performed on a Zeiss LSM700 confocal microscope.

### Statistical analysis

Quantifications were performed using data obtained from at least 3 replicate wells of fibroblasts or RPE cells, or 3 individually cultured organoids in separate wells of a 96-well plate, from 3 independent differentiation batches. Where used, at least 3 sections were averaged to account for regional variability in organoid differentiation. GraphPad Prism software v.8.0 was used for plotting graphs. One-way ANOVA analysis with Dunnett’s post hoc test within the software package was employed for statistical testing. p values are reported in figures. p <0.05 was considered statistically significant.

### Study approval

Skin biopsy donations were performed at the National Eye Institute Clinical Center under institutional review board-approved protocols 15-EI-0128 (ClinicalTrials.gov: NCT01432847) and 11-EI-0245 (ClinicalTrials.gov: NCT01432847).

## Author contributions

Conceptualization, A.S., K.K., Z.Q., and E.W.; methodology, investigation, validation, and analysis, K.K., Z.Q., E.W., H.S., S.H., and M.A.E.; resources, S.H., W.M.Z., and B.P.B.; writing – original draft, K.K., A.S., Z.Q., and E.W.; writing – review & editing, all authors; project administration, supervision, and funding acquisition, A.S.

## Data Availability

RNA sequencing (RNA-seq) data reported in this study are available at GEO: GSE200339. This paper did not generate original code. Any additional information required to reanalyze the data reported in this paper is available from the lead contact upon request. Further details of experimental procedures are provided in the supplemental information.

## Supplemental information

Document S1. Figures S1–S6, Tables S1–S3 and Supplemental experimental procedures

Document S2. Article plus supplemental information
