# Supplementary material for: In vitro modeling and rescue of ciliopathy associated with IQCB1/NPHP5 mutations using patient-derived cells
Source: Stem Cell Reports. 2022 Sep 8;17(10):2172–86. doi: 10.1016/j.stemcr.2022.08.006 (PMC9561628; doi:10.1016/j.stemcr.2022.08.006)
Supplement: Document S1. Figures S1–S6, Tables S1–S3 — and Supplemental experimental procedures [file mmc1.pdf]

Stem Cell Reports, Volume *17*

Supplemental Information

In vitro modeling and rescue of ciliopathy associated with *IQCB1/ NPHP5* mutations using patient-derived cells

Kamil Kruczek, Zepeng Qu, Emily Welby, Hiroko Shimada, Suja Hiriyanna, Milton A. English, Wadih M. Zein, Brian P. Brooks, and Anand Swaroop

In vitro modeling and rescue of ciliopathy associated with _IQCB1/NPHP5_ mutations using patient-derived cells

Kamil Kruczek, Zepeng Qu, Emily Welby, Hiroko Shimada, Suja Hiriyanna, Milton A. English, Wadih M. Zein, Brian P. Brooks, Anand Swaroop

Supplemental Dataset

Supplemental figures

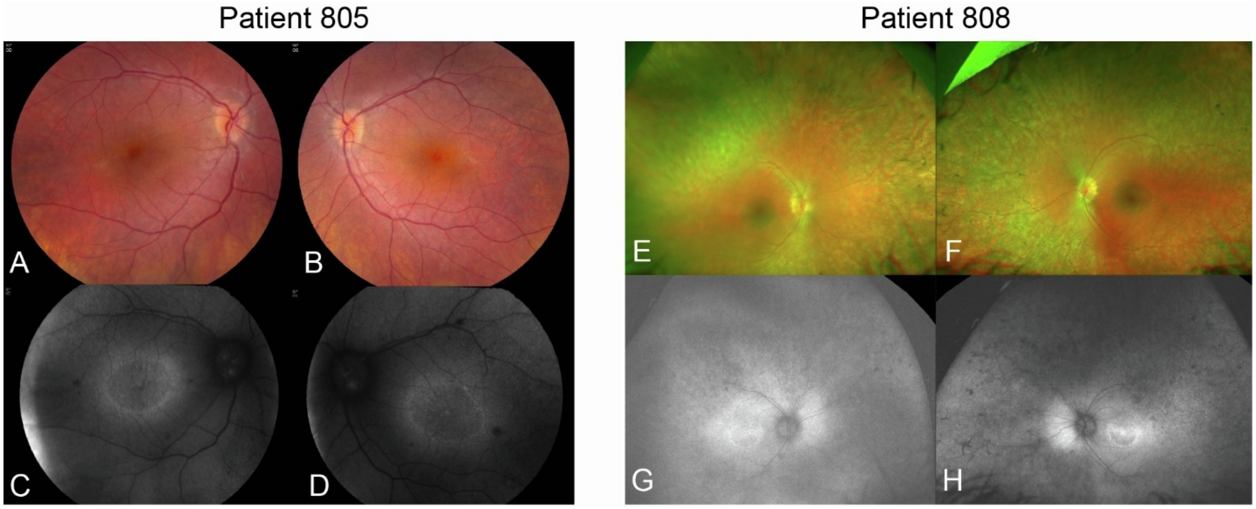

Figure S1. Representative fundus images of patients 805 and 808. Related to Figure 1.

Topcon color (A,B) and Optos wide-field pseudocolor fundus photography (E,F) and fundus autofluorescence (FAF) imaging (C,D,G,H) for patients 805 and 808 at the time of skin biopsy. The images document retinal findings of subtle pigment irregularities and moderate vascular attenuation in patient 805 (A-D). In patient 808 dark macular ring with advanced vascular attenuation, peripheral retinal pigment redistribution and bony spicules, and mild optic nerve pallor were observed (E-H). A ring of hyperautofluorescence surrounds the macula on FAF imaging with peripheral hypoautofluorescence corresponding to the retinal pigmentary changes. Note the blurry right eye images (patient 808) are due to corneal scarring caused by keratoconus (E,G). Of interest, the size of the hyperautofluorescent ring in both patients is similar despite the difference in the age at the time of testing. This likely indicates a more severe retinal degeneration phenotype in patient 805 (almost a decade younger than patient 808 at the time of imaging).

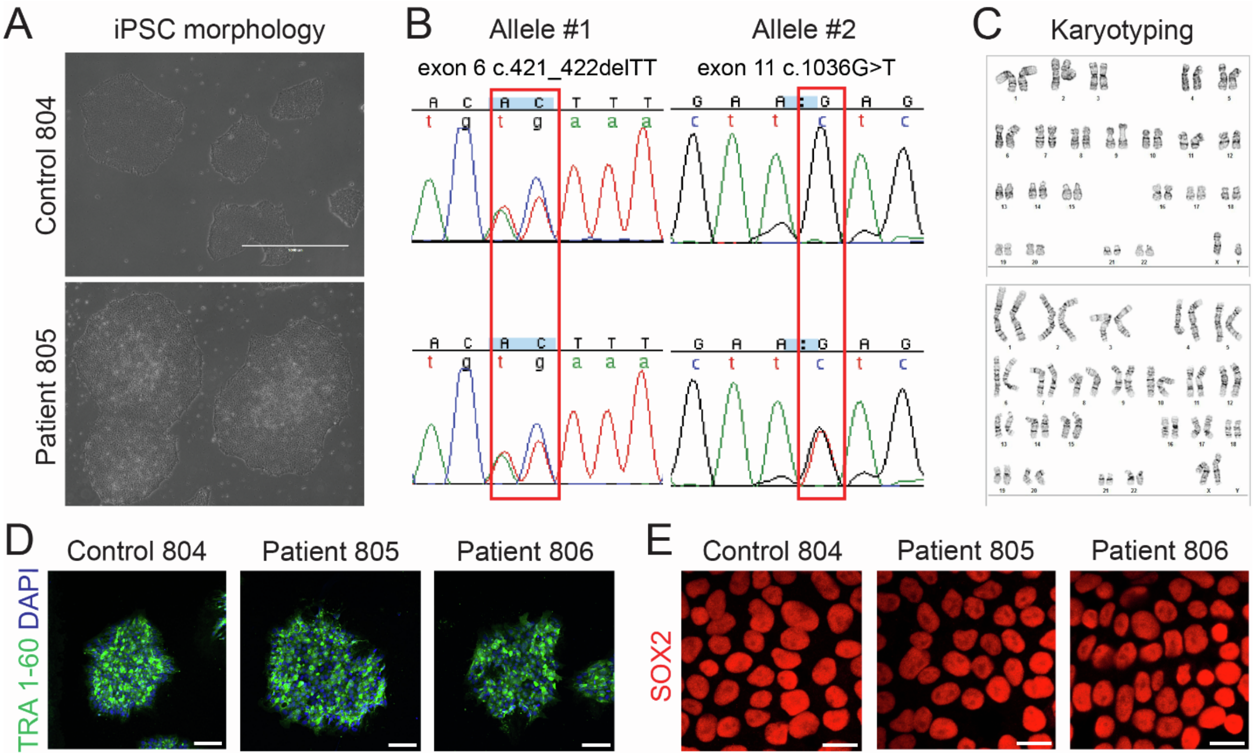

Figure S2. NPHP5-*LCA* iPSC lines. Related to Figures 2-6.

(A) Representative brightfield images show normal iPSC colony morphology of 804 control and 805 patient iPSC lines. **(B)** An example of Sanger sequencing from the two lines to validate *IQCB1*/*NPHP5* mutations, highlighted by red boxes. **(C)** Karyotyping was performed on iPSC lines to evaluate normal chromosome number and morphology. **(D,E)** Immunostaining of stem cell markers TRA-1-60 (D) and SOX2 (E) in 804 control and 805 and 806 NPHP5-LCA patient iPSC lines. Scale bars, 1000 μm in (A), 100 μm in (D), 20 μm in (E).

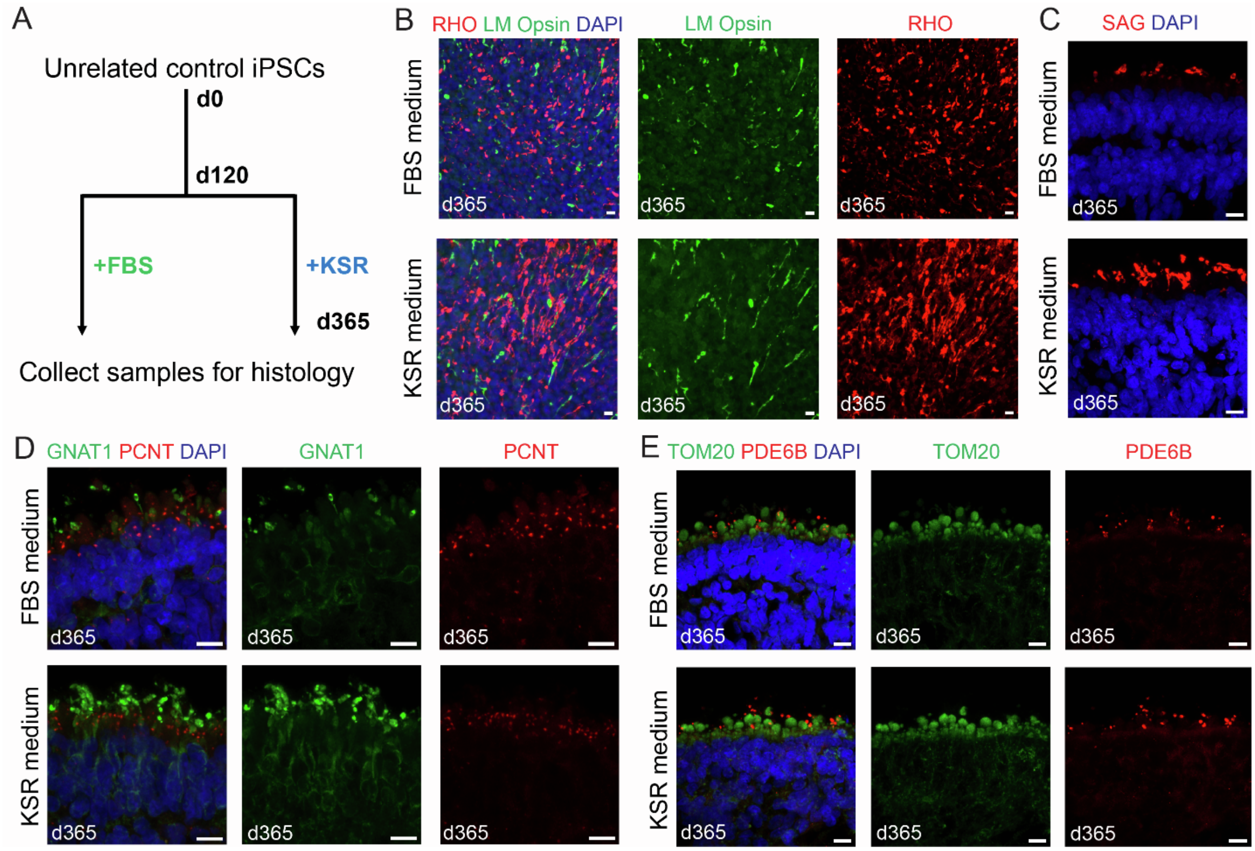

Figure S3. Serum free culture conditions support long-term maintenance of retinal organoids. Related to Figure 3.

(A) A schematic of the experimental design. **(B)** Wholemount staining of Rhodopsin (RHO) and L/M cone Opsins in organoids from unrelated control iPSC line at day 365 of differentiation. (C-E) Immunostaining of proteins associated with cilia, inner and outer segments of photoreceptors using cryosections. **(C)** Immunostaining of Visual Arrestin (SAG). **(D)** Co-staining of Rod Transducin (GNAT1) and ciliary basal body marker Pericentrin (PCNT). **(E)** Co-staining visualizing inner segments with a mitochondrial protein TOM20 and Phosphodiesterase 6B (PDE6B), which localizes to outer segments. Please note qualitative improvements in maintenance of outer segment structures in a long-term culture with use of medium containing knockout serum replacement (KSR) supplement rather than the fetal bovine serum (FBS). Scale bars, 10 µm.

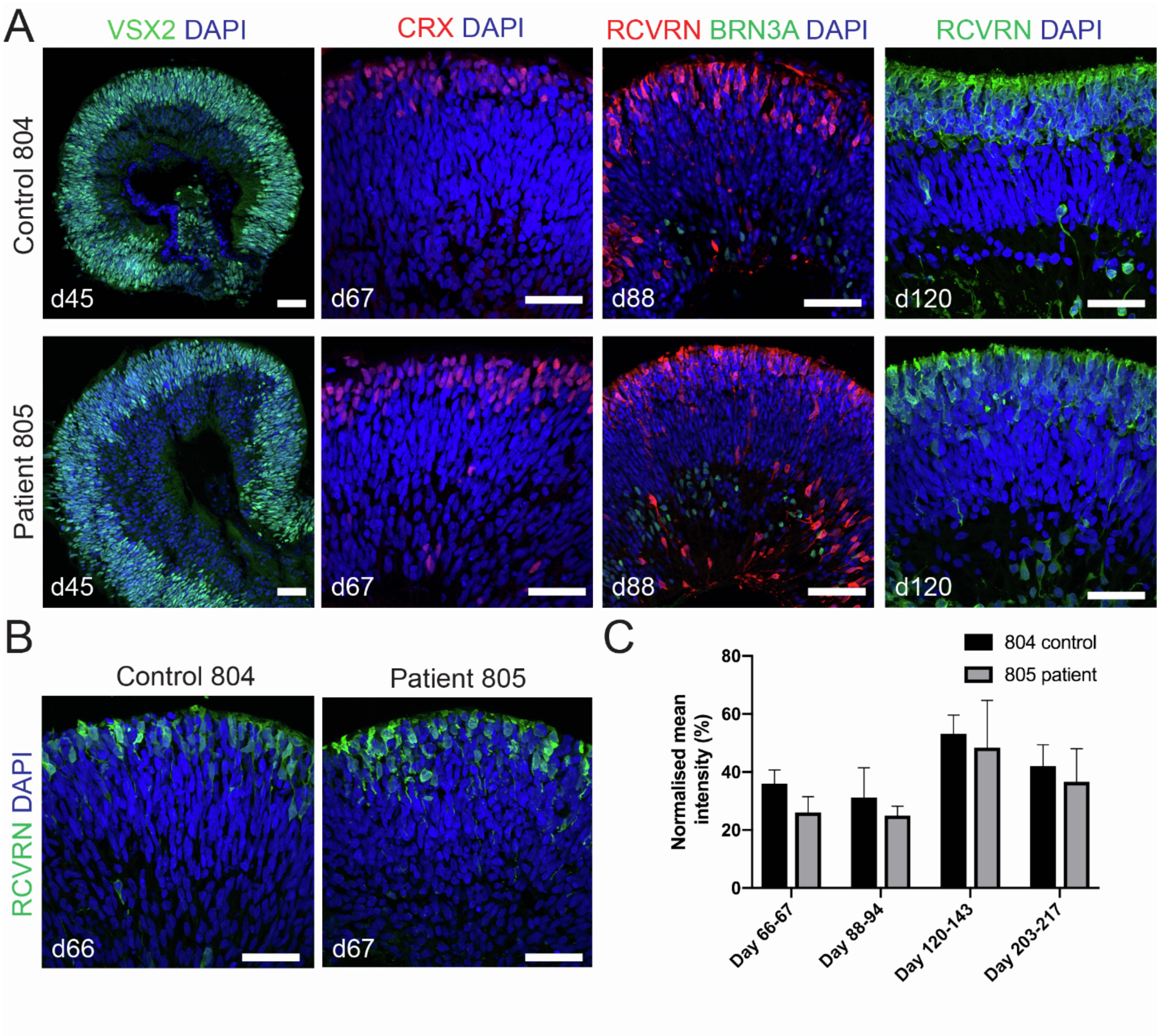

Figure S4. Differentiation of NPHP5-LCA retinal organoids. Related to Figure 3 and 4.

(A) Time course of retinal differentiation in NPHP5-LCA retinal organoids. Immunostaining in 804 control and 805 NPHP5-LCA patient retinal organoids for retinal progenitor marker VSX2 at d45 of differentiation, photoreceptor transcription factor CRX at d67, phototransduction-related protein Recoverin, and ganglion cell marker BRN3A at d88, and Recoverin at d120. Note the localization of Recoverin-positive developing photoreceptors to the apical aspect of neuroepithelium, in contrast to BRN3A-positive retinal ganglion cells aligning at the basal side at d88. **(B)** Staining for Recoverin at d66/67 in 804 control and 805 patient samples. **(C)** Quantification of Recoverin immunostaining intensity across differentiation time course. The data was obtained from at least 3 organoids at each time point, and with 3 sections averaged. No statistically significant differences were detected. Scale bars: 100 μm (in (A) Day 45 images), 50 μm (all other images).

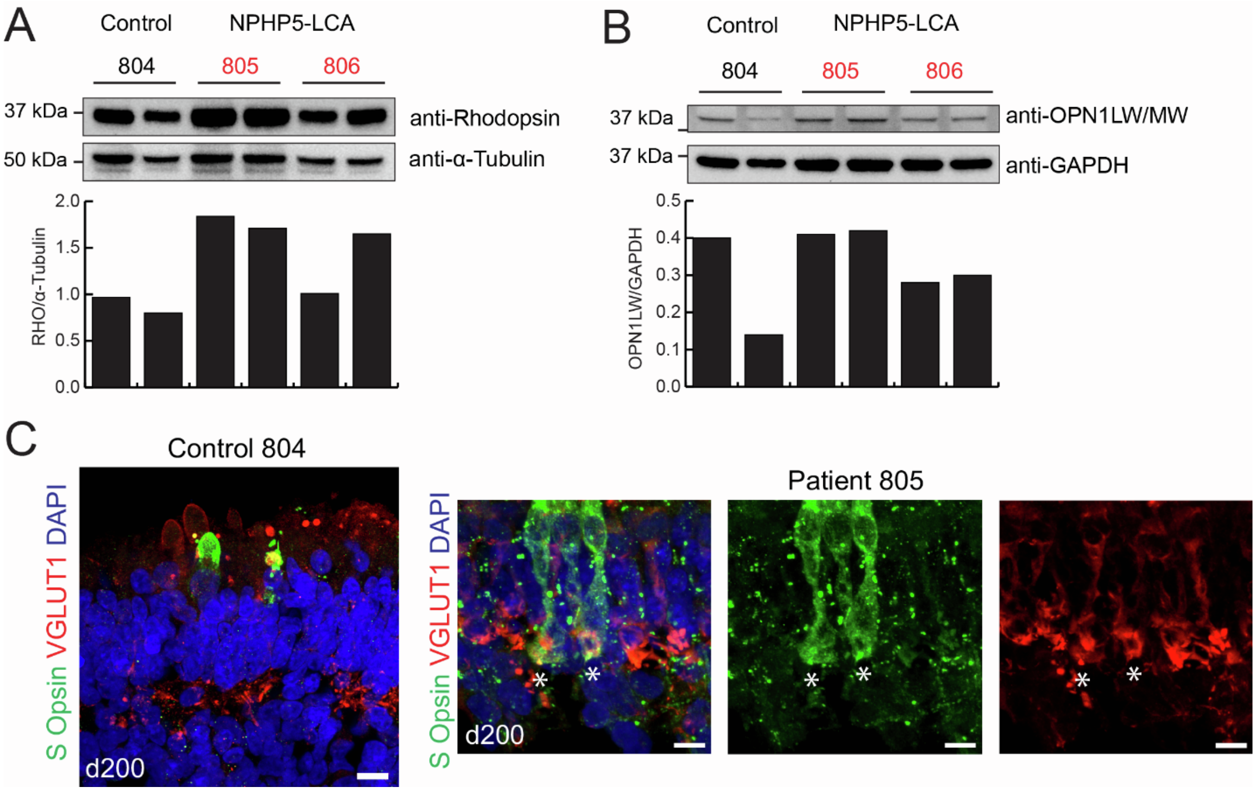

Figure S5. Opsin protein levels in NPHP5-LCA retinal organoids. Related to Figure 4.

Immunoblot analysis of protein extracts from control and NPHP5-LCA patient retinal organoids at day 200 of differentiation. Membranes were probed using antibodies against **(A)** Rhodopsin, or **(B)** Medium/Long wavelength-sensitive cone Opsins (OPN1L/MW). α-Tubulin or GAPDH were used as loading controls. Molecular mass markers (kDa) are indicated on the left. Two replicate samples from independent differentiation batches were used for the assay. Densitometry quantification of band intensity is presented in lower panels. **(C)** Immunolocalization of S Opsin and synaptic marker Vesicular Glutamate Transporter 1 (VGLUT1) in control and patient (805) retinal organoids at day 200 of differentiation. Nuclei counter stained with DAPI. Scale bar, 10 µm. Asterisks indicate mislocalization of S Opsin to axons and synaptic pedicles in patient organoids.

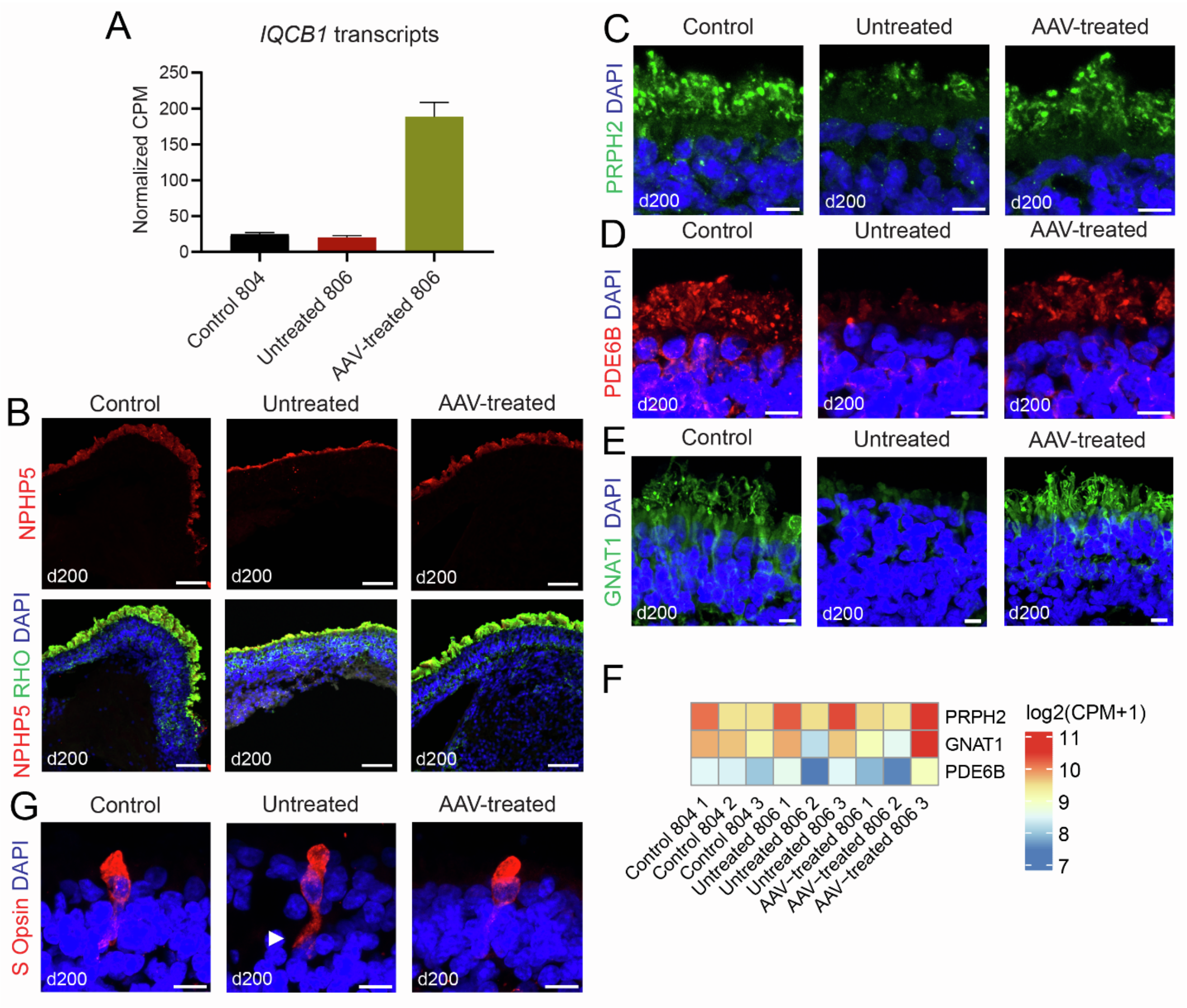

Figure S6. AAV-mediated *IQCB1*/*NPHP5* gene augmentation improves protein localization to photoreceptor outer segments. Related to Figure 6.

(A) *IQCB1* (NPHP5) mRNA expression in d200 organoids derived from 804 control, untreated 806, and 806 treated with AAV-NPHP5 vector at d120. Transcripts were quantified from RNA sequencing. CPM, counts per million reads. Data from 3 biological replicate samples in each group. **(B)** Staining of NPHP5 and Rhodopsin in sections from 804 control, untreated and AAV-treated 806 line organoids. Scale bars, 50 µm. **(C-E)** Immunostaining in cryosections from 804 control, untreated 806 and 806 line treated with AAV2-NPHP5 vector at day 120 of photoreceptor proteins localizing to outer segments at d200 of differentiation: **(C)** Peripherin2 (PRPH2), **(D)** Phosphodiesterase 6B (PDE6B) and **(E)** rod Transducin-alpha (GNAT1). **(F)** Heatmap of RNA-seq data from control 804, untreated and AAV-treated 806 patient retinal organoids at day 200 of differentiation. Values are shown as log_2(CPM+1). No significant changes were detected in the expression of selected genes. **(G)** Immunostaining of cone S Opsin visual pigment. Scale bar in all images in C-E and G, 10 µm.

Supplemental Tables

Patient ID	Disease	DNA variant of Allele #1	Protein variant of Allele #1	DNA variant of Allele #2	Protein variant of Allele #2	Sex	Age (yr) Diagnosis (Biopsy)
**NEI 801**	Unaffected control	Exon 8 c.659delC	p.S220X	Normal	Normal	F	- (40)
**NEI 802**	Patient	Exon 8 c.659delC	p.S220X	Exon 13 c.1362C>T	p.R455X	F	4 (11)
**NEI 804**	Unaffected control	Exon 6 c.421_422del TT	p.F141fsX6	Normal	Normal	M	- (50)
**NEI 805**	Patient	Exon 6 c.421_422del TT	p.F141fsX6	Exon 11 c.1036G>T	p.E346X	F	0.5 (8)
**NEI 806**	Patient	Exon 6 c.421_422del TT	p.F141fsX6	Exon 11 c.1036G>T	p.E346X	M	5 (10)
**NEI 807**	Unaffected control	Exon 13 c.1382C>T	p.R461X	Normal	Normal	F	- (52)
**NEI 808**	Patient	Exon 13 c.1382C>T	p.R461X	Exon 14 c.1516_1517 delCA	p.H506fsX13	M	3 (19)

Table S1. NPHP5-LCA patient information. Related to Figure 1.

Information on the recruited NPHP5-LCA families including details of *IQCB1/NPHP5* gene mutations. The last column of Age includes the age at initial diagnosis, with age at the time of biopsy given in parentheses.

Patient ID	NEI 802	NEI 805	NEI 806	NEI 808
BCVA OD	20/400	20/60	LP	20/1000
BCVA OS	20/500	20/50	LP	20/600
GVF V4e OD (horizontal, degrees)	60	5	Not seen	3
GVF V4e OS (horizontal, degrees)	80	5	Not seen	4
GVF I4e OD (horizontal, degrees)	Not seen	2	Not seen	Not seen
GVF I4e OD (horizontal, degrees)	Not seen	2	Not seen	Not seen
Nystagmus	Present	Present (upbeat)	Present	End-gaze
Strabismus	Intermittent Exotropia	Orthophoria	Exotropia	Intermittent Esotropia
Keratoconus	None	None	None	Present (with corneal scarring OD)
Macula	Normal Appearance	Subtle pigment irregularity	Xanthophyllic pigmentation	Dark macular ring
Retinal Vascular Attenuation	Mild	Moderate	Severe	Severe
Periphery	Granular	Subtle Pigment redistribution	RPE Mottling	Mottling and bony spicules
Optic Nerve Head Pallor	None	None	Mild	Mild
Kidney Disease	Normal (function and ultrasound)	Normal	Normal	Elevated BUN and Cr; moderate decrease in eGFR

Table S2. Clinical presentation of patients carrying *IQCB1/NPHP5* mutations. Related to Figure 1.

Summary of findings at the time of clinical examination of patients with *IQCB1/NPHP5* mutations. Please note that, given the young age of patients in the study, kidney dysfunction may still occur later in life altering the diagnosis from LCA to SLSN. BCVA – Best Corrected Visual Acuity, GVF – Goldmann Visual Field, BUN – Blood Urea Nitrogen, Cr – Creatinine, eGFR – estimated Glomerular Filtration Rate

Antigen	Species/type	Dilution	Source	Identifier	Application
Actin Beta	Mouse monoclonal	1:1000	Millipore-Sigma	A5316	WB
ARL13B	Rabbit polyclonal	1:250	Proteintech	17711-1-AP	IHC
BRN3A	Mouse monoclonal	1:100	Millipore-Sigma	MAB1585	IHC
CEP290	Rabbit polyclonal	1:1000	Proteintech	22490-1-AP	WB
CRX	Mouse monoclonal	1:100	Abnova	H00001406-M02	
GAPDH	Mouse monoclonal	1:1000	Millipore-Sigma	G8795	WB
GNAT1	Mouse monoclonal	1:200	Santa Cruz Biotech	sc-136143	IHC
GT335	Mouse monoclonal	1:250	AdipoGen	AG-20B-0020	IHC
IFT88	Rabbit polyclonal	1:100	Proteintech	13967-1-AP	IHC
L/M Opsin	Rabbit polyclonal	1:250	Millipore	AB5405	IHC
NPHP5	Goat polyclonal	1:100	Santa Cruz Biotech	sc-51343	IHC
PDE6B	Rabbit polyclonal	1:200	Custom Tiansen Li	n.a.	IHC
Pericentrin	Rabbit polyclonal	1:250	Abcam	ab4448	IHC
Peripherin2	Chicken polyclonal	1:200	Custom Tiansen Li	n.a.	IHC
Phalloidin	Alexa 488-conjugated	1:400	ThermoFisher Scientific	A12379	IHC
PMEL17	Mouse monoclonal	1:200	Novus Biologicals	NBP2-44520	IHC
Recoverin	Rabbit polyclonal	1:500	Chemicon International	AB5585	IHC
Rhodopsin	Mouse monoclonal	1:500	Custom Robert Molday	Clone 1D4	IHC
Visual Arrestin	Mouse monoclonal	1:500	Abcam	ab190315	IHC
VSX2	Sheep polyclonal	1:200	Abcam	ab16142	IHC
SOX2	Rabbit polyclonal	1:100	STEMGENT	09-0024	IHC
S Opsin	Rabbit polyclonal	1:250	Millipore-Sigma	AB5407	IHC
TOM20	Mouse monoclonal	1:250	Santa Cruz Biotech	sc-17764	IHC
TRA-1-60	Mouse monoclonal	1:200	Millipore-Sigma	MAB4360	IHC
Tubulin Acetylated	Mouse monoclonal	1:500	Millipore-Sigma	T6793	IHC
Tubulin Alpha	Mouse monoclonal	1:1000	Abcam	Ab7291	WB
Tubulin Gamma	Mouse monoclonal	1:500	Millipore-Sigma	T6557	IHC

Table S3. List of antibodies and related reagents used in the study. Related to all figures.

Antibodies used in the study, including information on type, source, dilution and application used; IHC – immunostaining, WB – immunoblotting.

Experimental Procedures

Ophthalmology Clinical Assessment

Patients participated in detailed ophthalmic assessment including measurement of best-corrected visual acuity (BCVA), Goldmann visual field testing, slit-lamp biomicroscopy, fundus exam, and electrodiagnostics (when able). Clinical imaging documented retinal phenotype at time of biopsy with Optos wide-field imaging (Dunfermline, Scotland) to obtain both pseudocolor images and fundus autofluorescence. Details of the approach to examine patients with ciliopathies are published in a description of a large Joubert syndrome cohort (Brooks et al., 2018). A similar approach was followed in this study.

Isolation of Fibroblasts from Skin Biopsies

Human fibroblasts were isolated from healthy individual and NPHP5 patient skin biopsies using similar methods as previously described in (Shimada et al., 2017). Briefly, skin biopsies were cultured overnight in DMEM (Gibco) containing 0.1% trypsin/EDTA (Gibco) to facilitate the removal of the epidermis. Once removed, biopsies were dissected into small pieces (1-2mm^2) and plated on to gelatin-coated 6 well plates with fibroblast media (DMEM or αMEM (Gibco), 10% FBS (Gibco) and 1x penicillin/streptomycin (Gibco) to promote fibroblast outgrowth. Media was changed every 3 days and cells were cultured at 37°C, 5% CO_2. Once fibroblasts had reached 80-90% confluency, the cells were dissociated in TrypLE Express (Gibco) for 5 min at 37°C, before the addition of fibroblast media and centrifugation at 300 x g for 5 min. Cells were replated into T25 cm^2 tissue culture flasks and were expanded to make frozen stocks, for iPSC reprogramming and for ciliogenesis assays.

iPSC Reprogramming from Fibroblasts

Fibroblasts were reprogrammed into iPSC lines by the NIH/NHLBI iPSC and Genome Engineering Core Facility using the integration free Sendai virus method as previously described in (Beers et al., 2015).

Ciliogenesis Assay

Fibroblasts were seeded on to collagen-coated chamber slides (μ-Slide 8 well; ibidi) at a density of 10,000-20,000 cells per chamber. Once cells had reached 90-100%, fibroblasts were serum starved (FBS withdrawal from medium) for 72 hr to promote cilia formation. Cells were then fixed in 4% PFA for 10 min at room temperature, before washing with 1x PBS and proceeding to immunocytochemistry experiments.

Quantitative RT-PCR

RNA was isolated from frozen samples using RNeasy Mini Kit (Qiagen) according to manufacturer's instructions. Concentration of purified RNA was determined using Nanodrop spectrophotometer (ThermoFisher Scientific). One μg of RNA per sample was used to prepare cDNA using QuantiTect Reverse Transcription Kit following manufacturers protocol. 20 ng of cDNA was then used for a single qPCR reaction using PowerUp SYBR Green Master Mix (Applied Biosystems). *IQCB1* (NPHP5) transcripts were amplified using following primer sequences:
Forward sequence: 5'-GCACCAACGTTGAACAGCTA-3'
Reverse sequence: 5-'CTCCAAGCTTCTTCCACCAG-3'
ACTB (Actin beta) was used as a housekeeping gene for normalization amplified with primer sequences:
Forward sequence: 5'-ACAGAGCCTCGCCTTTGCC-3'
Reverse sequence: 5'-GATATCATCATCCATGGTGAGCTGG-3'

The reactions were run on 7900 HT Fast Real-Time PCR System7900 HT Fast Real-Time PCR System (Applied Biosystems). The results were analyzed using ddCt method (Livak and Schmittgen, 2001) in Microsoft Excel software.

Immunostaining

Immunostaining was performed as previously described (Kruczek et al., 2021). Briefly, frozen organoid cryosections were thawed at room temperature and rehydrated using PBS for 15 min. Sections (or chamber slides) were blocked with a solution of 1% BSA (Sigma-Aldrich), 0.1% Triton X-100 (Sigma-Aldrich) and 5% donkey or goat serum (ThermoFisher Scientific) in PBS for 2 hr at room temperature. Primary antibodies were added overnight in 1% BSA, 0.1% Triton X-100 in PBS. In the morning, samples were washed 5x with PBS. Alexa Fluor-conjugated secondary antibodies (Invitrogen) were added at 1:200 dilution for 2 hr at room temperature in 1% BSA, 0.1% Triton X-100 in PBS. Sections were washed 4× with PBS, then incubated with 4',6-diamidino-2-phenylindole (DAPI) for 15 min, before mounting with Fluoromount-G mounting medium (SouthernBiotech) and covering with a microscopy cover glass (VWR). Imaging was performed on Zeiss LSM700 confocal microscope using ZEN Blue software (Zeiss) for acquisition and initial processing. Images were further processed using Adobe Photoshop (Adobe) and ImageJ software packages. Quantifications were performed in ImageJ software (Schroeder et al., 2021).

Immunoblotting

Protein extracts were prepared in RIPA buffer. Protein amount was quantified using BCA assay (ThermoFisher Scientific). Extracts were boiled for 5 minutes at 95°C then loaded onto precast 12% polyacrylamide Mini-PROTEAN gels (Bio-Rad). Wet transfer was performed overnight at 25V onto a PVDF membrane. Membrane was then blocked using 5% milk in TBS-T buffer for 1 hr at room temperature with agitation. Primary antibody was added overnight in TBS-T buffer at 4°C. Membrane was washed 3× with TBS-T for 5 min. HRP-conjugated secondary antibodies were added at 1:1000 dilution in TBS-T buffer for 2 hours at room temperature, followed by 3 washes with TBS-T buffer, 5 min each. Membrane was transferred to PBS. Bands were visualized using SuperSignal West Pico substrate solution (ThermoFisher Scientific) and detected on Bio-Rad ChemiDoc imager (Bio-Rad).

Production of AAV Vectors

Human *IQCB1/NPHP5* coding sequence was cloned from a Myc-tagged NPHP5 expression construct kindly provided by Dr Wolfgang Baehr at University of Utah and placed upstream of CMV promoter in an pV5.2 AAV vector backbone. Resulting viral vector was prepared following a previously described protocol (Kruczek et al., 2021; Yu and Wu, 2021) using $CaCl_2$-mediated transfection of helper, capsid and transgene plasmid into HEK293 cell line. AAV particles were purified from cell lysate using ultracentrifugation on CsCl gradient and dialysis. Titer of vector preparations was determined using a qPCR assay. First, two independent dilutions (100×, 300×) of samples were prepared in qPCR dilution buffer (10mM Tris pH 8.0, 1 mM EDTA, 10 µg/ml yeast RNA, 0.01% Tween 80). Then, 10 µl of diluted sample was added to 40 µl of DNase solution, containing 39 µl of digestion buffer 10 mM Tris pH 8.0, 10 mM $MgCl_2$ and 1 µl DNase (10 U), and incubated at 37°C for 1 hr. 50 µl of 200 mM EDTA, pH 8.0 was added, samples mixed and incubated at 95°C for further 30 min. Next, samples were diluted 100x by transferring 10 µl of sample to 990 µl qPCR dilution buffer, vortexed thoroughly and 5 µl of resulting diluted solution used for PCR reaction. TaqMan Fast Universal PCR Master Mix (Applied Biosystems) was used for PCR reaction. *IQCB1/NPHP5* vector contains CMV promoter, which was used to quantify viral genomes. CMV promoter sequence was amplified using primer sequences:

Forward 5'-TGGGAGTTTGTTTTGCACCAA-3',
Reverse 5'-CGCCTACCGCCCATTTG-3',
and a probe used for qPCR reaction:

CMV probe 5'-6FAM-TCCAAAATGTCGTAACAACT–MGBNFQ-3'

The final concentration of each primer was 900 nM, and the final concentration of the probe 250 nM. The reactions were run on 7900 HT Fast Real-Time PCR System7900 HT Fast Real-Time PCR System (Applied Biosystems). Titer was determined using original vector plasmid DNA standard. Quantity of viral genomes was calculated based on dilution of original sample (10^5 or 3×10^5 fold) with values multiplied by 2×10^7 or 6×10^7 to give vector genomes per ml.

AAV Transduction of Retinal Organoids

Individual retinal organoids were cultured in ultra-low attachment 96-well plates (Sbio). At day 120, differentiation medium (3:1 neural induction medium supplemented with 10% FBS and 500 nM 9-*cis* retinal) was aspirated, AAV vector preparation diluted in culture medium to 10^{11} viral genomes per organoid to a total of 100 µl per organoid and added to the selected wells. The following day, the medium was changed to regular differentiation medium and organoids cultured as before.

RNA Sequencing

RNA-seq and data analysis were performed as described (Kruczek *et al*., 2021). In brief, total RNA was extracted from 3 frozen organoids per sample using RNeasy Mini kit (Qiagen). Purified RNA samples with RNA integrity number (RIN) of higher than 7 were used for library generation by the TruSeq Library Preparation Kit (Illumina). Paired-end sequencing was performed to 125 bases on Illumina HiSeq 2500. The alignment and quantification pipeline used human reference genome GRCh38.p7 and Ensembl v.94 for annotation. Transcript-level counts were performed by kallisto v0.45.0 package and summarized to gene level. Gene level counts were converted to count per million (CPM) and then TMM normalized using edgeR v.3.32.0 package in R (v.4.0.3)/Bioconductor environment.

References

Beers, J., Linask, K.L., Chen, J.A., Siniscalchi, L.I., Lin, Y., Zheng, W., Rao, M., and Chen, G. (2015). A cost-effective and efficient reprogramming platform for large-scale production of integration-free human induced pluripotent stem cells in chemically defined culture. Sci Rep *5*, 11319.

Brooks, B.P., Zein, W.M., Thompson, A.H., Mokhtarzadeh, M., Doherty, D.A., Parisi, M., Glass, I.A., Malicdan, M.C., Vilboux, T., Vemulapalli, M.*, et al.* (2018). Joubert Syndrome: Ophthalmological Findings in Correlation with Genotype and Hepatorenal Disease in 99 Patients Prospectively Evaluated at a Single Center. Ophthalmology *125*, 1937-1952.

Kruczek, K., Qu, Z., Gentry, J., Fadl, B.R., Gieser, L., Hiriyanna, S., Batz, Z., Samant, M., Samanta, A., Chu, C.J.*, et al.* (2021). Gene Therapy of Dominant CRX-Leber Congenital Amaurosis using Patient Stem Cell-Derived Retinal Organoids. Stem Cell Rep *16*, 252-263.

Livak, K.J., and Schmittgen, T.D. (2001). Analysis of relative gene expression data using real-time quantitative PCR and the 2(-Delta Delta C(T)) Method. Methods (San Diego, Calif) *25*, 402-408.

Schroeder, A.B., Dobson, E.T.A., Rueden, C.T., Tomancak, P., Jug, F., and Eliceiri, K.W. (2021). The ImageJ ecosystem: Open-source software for image visualization, processing, and analysis. Protein Sci *30*, 234-249.

Shimada, H., Lu, Q., Insinna-Kettenhofen, C., Nagashima, K., English, M.A., Semler, E.M., Mahgerefteh, J., Cideciyan, A.V., Li, T., Brooks, B.P.*, et al.* (2017). In Vitro Modeling Using

Ciliopathy-Patient-Derived Cells Reveals Distinct Cilia Dysfunctions Caused by CEP290 Mutations. Cell Rep *20*, 384-396.

Yu, W., and Wu, Z. (2021). Ocular delivery of CRISPR/Cas genome editing components for treatment of eye diseases. Adv Drug Delivery Rev *168*, 181-195.
